# ﻿Three new species of *Polycarpaea* (Caryophyllaceae) from Kerala, South India

**DOI:** 10.3897/phytokeys.213.89875

**Published:** 2022-11-14

**Authors:** Sindhu Arya, Venugopalan Nair Saradamma Anil Kumar, Ambika Viswanathan Pillai, Alex Philip Alen, Jose Sojan, Veerankutty Suresh

**Affiliations:** 1 Department of Botany, University College, University of Kerala, Thiruvananthapuram, Kerala– 695 034, India University of Kerala Thiruvananthapuram India; 2 Department of Botany, Government Victoria College, University of Calicut, Palakkad, Kerala– 678001, India University of Calicut Palakkad India; 3 Department of Botany, Government College, Chittur, Palakkad, Kerala– 678104, India Department of Botany, Government College Palakkad India

**Keywords:** Caryophyllales, Palakkad gap, *Polycarpaea*, Western Ghats

## Abstract

Three new species of *Polycarpaea*, *Polycarpaeabarbellata*, *P.ebracteata* and *P.psammophila*, are described from the Palakkad district of Kerala, India. The new species are allied to *P.corymbosa* and *P.aurea* but can be visibly distinguished by unique character combinations, *viz.* shape of sepal, petal, bract and bracteole and seed morphology. Detailed descriptions along with illustrations and photographs are provided.

## ﻿Introduction

The genus *Polycarpaea*Lamarck (1792: 3) (Caryophyllaceae Juss.) comprises approximately 50 species which are mostly distributed in the tropics and subtropics of the old world and a few occur in the New World tropics (Dequan and Gilbert 2001; Mabberley 2008). The genus is represented in India by seven species (Arya et al. 2021).

During the field exploration carried out as part of the floristic studies of the southern Western Ghats in the Kerala region, several specimens of morphologically unique *Polycarpaea* were collected from the hillocks of Palakkad district (Northern Kerala, India). On the basis of critical evaluation of collected specimens, comparison with various herbaria and through literature review, we found that these specimens are distinct from all other known species. Hence, we propose them as novel species.

## ﻿Materials and methods

Forest exploration trips were carried out during the period of June–January of 2020–21. Herbarium specimens of collected plants were deposited in the Herbarium UCBD. Additional herbarium specimens were examined from the Herbaria E, MH, K, TBGT, UCBD (acronyms according to Thiers 2022 [continuously updated]). Relevant literatures were analyzed (Wight 1843, 1850; Edgeworth and Hooker 1874; Dunn 1915; Majumdar 1993; Daniel et al. 2000; Venu et al. 2001; Daniel 2005; Mastakar et al. 2015; Geethakumary et al. 2019). A total of more than 50 flowers from each species were assessed to confirm the consistency of traits in the collected specimens and to validate the character occurrence.

## ﻿Results and discussion

### ﻿ Polycarpaea ebracteata

Taxon classificationPlantaeCaryophyllalesCaryophyllaceae

S. Arya, V.S.A. Kumar, V. Suresh & Alen Alex sp. nov.

53C7669D-1BBD-5531-B17A-352057B3DEBF

urn:lsid:ipni.org:names:77307989-1

Figs 1 , 2

#### Type.

India. Kerala, Palakkad district, Kollengode forest range, Nenmeni, Vengappara 10°34'33.6"N, 76°42'47.1"E, 160 m a.s.l., 20 September 2021, Suresh V., V.S.A. Kumar & Arya S., 2077 (holotype UCBD! isotype UCBD!).

**Figure 1.**
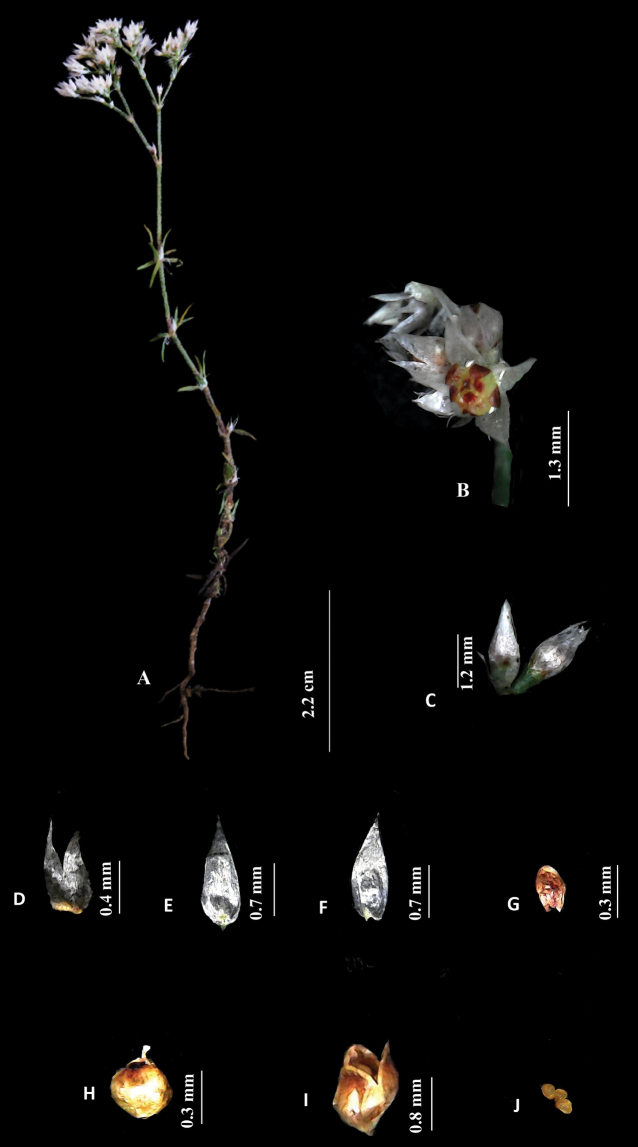
*Polycarpaeaebracteata***A** habit **B** flower **C** flower bud **D** stipule **E, F** sepal **G** petal **H** gynoecium **I** capsule **J** seed. Photos by Arya Sindhu.

#### Diagnosis.

*Polycarpaeaebracteata* is morphologically similar to *Polycarpaeacorymbosa* in terms of having prominent villous stem nodes and shape of petals but differs with respect to stipules (short, ovate less than 1mm, *vs.* lanceolate, long, 5 mm) bracts (absent *vs.* present), bracteoles (absent *vs.* present), sepals (ovate, acute at apex, not membranous *vs.* lanceolate, acuminate apex, hyaline, membranous), petals (dark brown, ovate-elliptical *vs.* whitish-pink, broadly ovate), stamens (filament reduced, 0.1 mm *vs.* filaments equal to the length of anther 1 mm), capsule (style not persistent, tips curved *vs.* style persistent, tips not curved) and seeds (3–4 or rarely 2, yellow, ovate *vs.* 5–13, brown, reniform).

**Figure 2.**
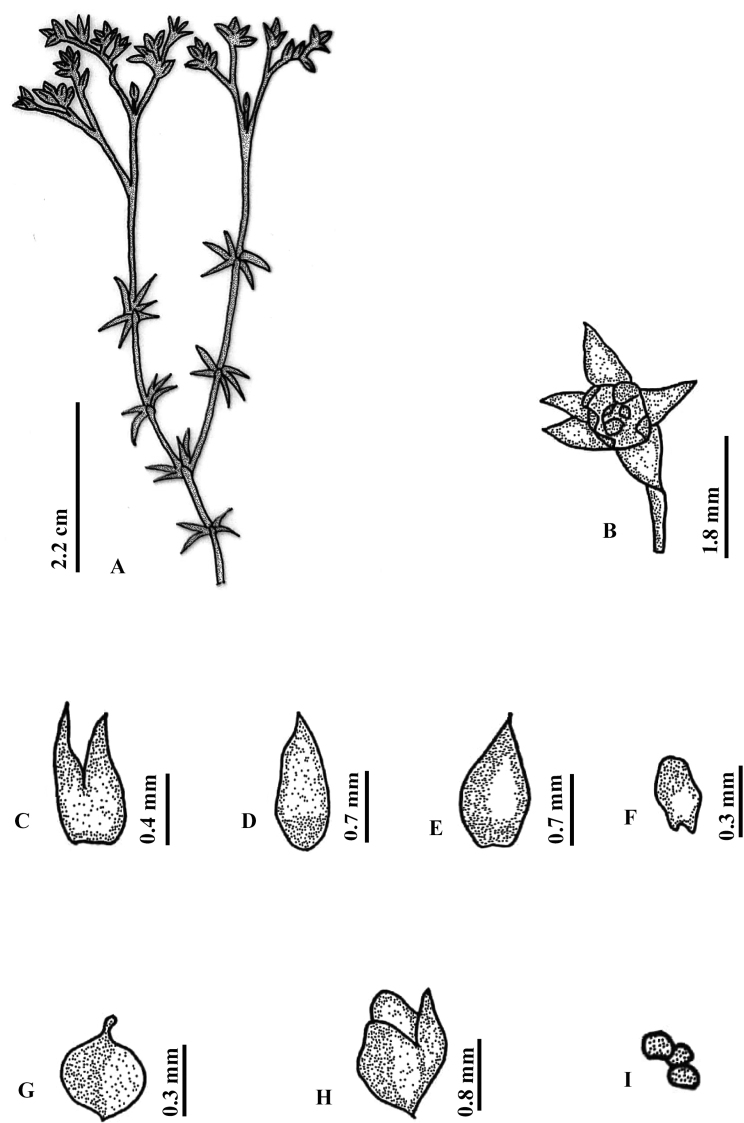
*Polycarpaeaebracteata***A** habit **B** flower **C** stipule **D, E** sepal **F** petal **G** gynoecium **H** capsule **I** seed. Illustration by Ambika Viswanathanpillai.

#### Description.

Annual herbs, erect or sub-erect, branched at base, 2.5–8 cm high. Stem terete, densely villous, nodes green, swollen, internodes ca. 5 mm long. Leaves whorled or verticillate, sessile, linear, green, 0.7–0.9 cm long, base cordate, margins smooth, apex acute or acuminate, surface glabrous, blade 1-veined, prominent on abaxial side; stipules scarious, ovate-obovate, (0.2–0.8 × ca. 0.6 mm), margins entire, acute, not nerved, yellowish or greenish at the base, white above. Inflorescence terminal, branched cyme, ca. 1.0 cm long; Flowers 2.2–2.6 mm long; Bracts absent. bracteoles absent; pedicels 1.0–1.3 mm long, green, villous. Sepals 5, free, ovate (1.3–1.4 × ca. 0.7 mm), entire at the margin, acute or obtuse at apex, white, non-membranous base round, midrib not prominent. Petals 5, ovate (0.1–0.3 × 0.1–0.3 mm), margins entire, oblong to round at apex, partially enclosing the ovary, 1/4 as short as sepals, dark red-brown. Stamens 5, forming a ring with petals and encircling the ovary, ca. 0.2 mm long; anthers yellow, oblong, basifixed. Ovary 1-loculed, shortly stipitate, spheroidal, 0.3–0.5 × 0.1–0.2 mm, glabrous, placentation free central; style 0.08–0.1 mm, shorter than the ovary, slender; stigma capitate. Capsule ovoid (1.4–1.6 × ca. 0.6 mm), shortly stipitate, 3-valved, breaks along the suture, brownish, scarious along margin. Seeds 3–4 (rarely 2), ovate (0.2–0.3 × 0.1–0.2 mm), yellow with no striations.

Micromorphology of the seed shows that it is round-oblong with a winged margin. The surface has sub parallel striations which are prominent. The striations do not cross each other and the encircling surface of the striations are punctate. Along the margins, the surface has parallel striations (Fig. 7E, F).

#### Etymology.

Latin prefix e-, without, bractea, bract, and suffix -ata, possession, alluding to absence of bracts, a diagnostic character.

#### Phenology.

Flowering and fruiting during August – December

#### Distribution and habitat.

The primary habitat of *Polycarpaeaebracteata* is the hillock terrains in Palakkad district (Granite outcrop in the southern side of Palakkad gap, the largest break in the Western Ghats having an arid climate with seasonal fires, in the state of Kerala). One of the common species that emerges after the initial rain are members of the Genus *Polycarpaea*, especially *Polycarpaeaaurea* (Wight 1850: 44) Dunn (1915: 65). *Polycarpaeaebracteata* is seen associated with *Allmanianodiflora* (L.) R. Br. ex Wight, *Indigoferaaspalathoides* DC. and *Fimbristyliscymosa* R. Br. (Fig. 8).

#### Conservation status.

The present study could report only three populations each with 15–20 individuals. Since *Polycarpaeaebracteata* could occur in further sites in SW-India (and India as a whole), we think that further data is required to ascertain the conservation status of the new taxon. As a consequence, the new species is here assessed as DD (Data Deficient) according to the IUCN criteria (IUCN 2021).

#### Additional specimens examined.

*Polycarpaeaebracteata* India. Kerala, Palakkad district, Kollengode, Cheerani. 12 September 2021, Suresh V. & Alen Alex Philip, 2061 (UCBD!); 20 September 2021, Sojan Jose & Suresh V, 2078 (UCBD!).

### ﻿ Polycarpaea psammophila

Taxon classificationPlantaeCaryophyllalesCaryophyllaceae

V. Suresh, V.S.A. Kumar, S. Arya, & Alen Alex sp. nov.

3A14FFF5-04FC-5DC4-905B-D583BF89FF39

urn:lsid:ipni.org:names:77307990-1

Figs 3 , 4

#### Type.

India. Kerala, Palakkad district, Nenmara, Ayinampadam, 10°35'29.4"N, 76°34'48.2"E, 140 m a.s.l., 21 September 2021, Suresh V. & Arya S., 2081 (holotype UCBD! isotype UCBD!).

**Figure 3.**
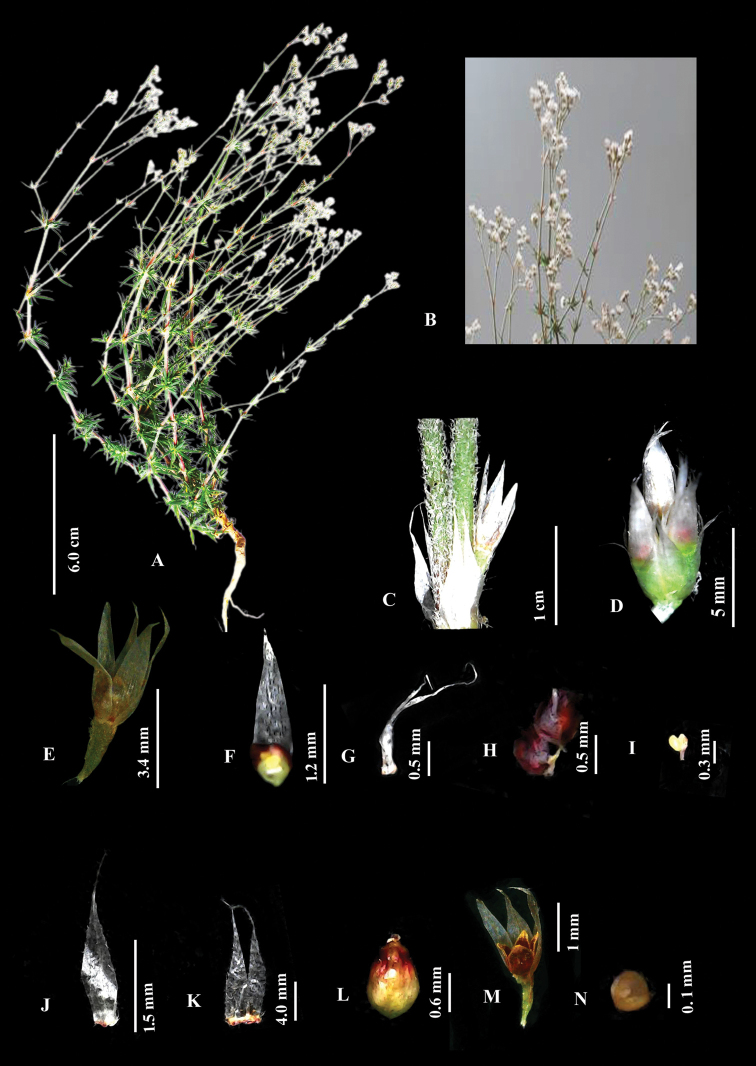
*Polycarpaeapsammophila***A** habit **B** inflorescence **C** internode **D** flower cluster **E** flower **F** bract **G** bracteole **H** petal **I** stamen **J** sepal **K** stipule **L** gynoecium **M** capsule **N** seed. Photos by Suresh V.

#### Diagnosis.

*Polycarpaeapsammophila* is morphologically similar to *Polycarpaeacorymbosa* with respect to the whorled arrangement of leaves and pilose nature of stem but differs with respect to stipules (linear to lanceolate with acuminate apex *vs.* lanceolate-ovate, with acute apex), bract (lanceolate-oblanceolate white, exceeding the length of sepal *vs.* lanceolate-ovate, shorter than the sepal), bracteoles (linear with acicular apex *vs.* lanceolate with acute apex), petals (ovate – oblate, keeled, dark brown, apex pointed upwards *vs.* broadly ovate, not keeled, whitish-pink, round at apex), gynoecium (oblate spheroidal, reddish yellow *vs.* ovate short, green), capsule (four valved *vs.* three valved) and seeds (20–25 yellowish brown, ovate *vs.* 5–13, brown, reniform).

**Figure 4.**
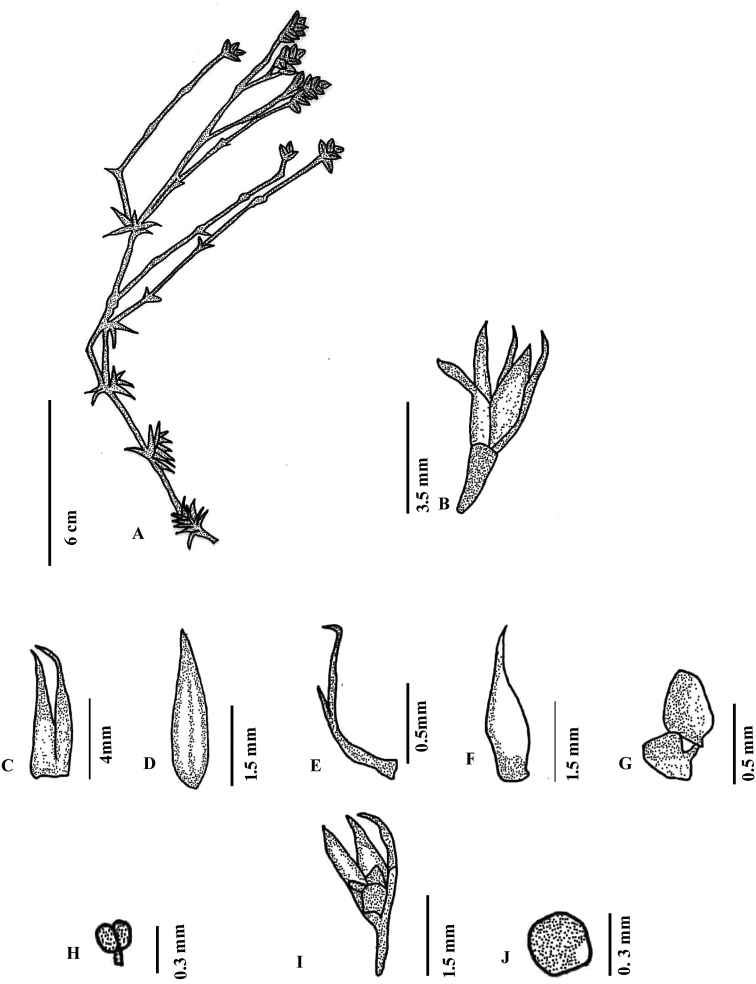
*Polycarpaeapsammophila***A** habit **B** flower **C** stipule **D** bract **E** bracteole **F** sepal **G** petal **H** stamen **I** capsule **J** seed. Illustration by Ambika Viswanathanpillai.

#### Description.

Annual herbs, erect or sub-erect, 18–25 cm high. Stems terete, densely villous, nodes green, swollen, internodes ca. 1.5–2 cm long. Leaves whorled, sessile, linear-lanceolate, green, 2.3–3.1cm long, base round, margin smooth, daggered in young leaf, apex acute or obtuse abaxial surface glabrous, adaxial surface pubescent along the mid vein; blade 1–2 veined, prominent on abaxial side; stipules prominent, linear to lanceolate, fused at the base (5–8 × ca. 2 mm), base golden yellow with unicellular setae; setae hyaline; margins entire, often bifurcated into two, branches acicular at apex, not nerved, milky white. Inflorescence terminal, irregular, branched lax cyme, ca. 10 cm long; Bracts lanceolate-oblanceolate, exceeding the length of the sepal (2.0 – 2.3 × ca. 0.3 mm); base smooth, margin entire, apex acuminate. Bracteole 1.3mm linear with acicular apex, holding the bracts in position. Flowers 8–10 per cyme, 4–5.5 mm long; pedicels 1.0–1.3 mm long, green villous. Sepals 5, free, obovate-oblanceolate (2.3–2.6 × ca. 0.7 mm), entire at the margin, acute or obtuse at apex, white, non-membranous base round, midrib faint. Petals 5, ovate-oblate (1–1.3 × 1.1–1.3 mm), margin entire, keeled, pointed upward at apex, partially or completely enclosing the ovary, 1/2 as short as sepals, dark red-brown. Stamens 5, forming a ring with petals and encircling the ovary, ca. 0.3 mm long; anthers yellow, oblong, basifixed. Ovary 1-loculed, shortly stipitate, spheroidal, reddish-yellow 1.2–1.3 × 1–2 mm, glabrous, placentation free central; style 0.2–0.3 mm, shorter than the ovary, often very reduced and slender; stigma capitate. Capsule oblate-prolate (1.4–1.6 × ca. 0.6 mm), style persistent, shortly stipitate, 4-valved, breaks along the suture, brownish, scarious along margin. Seeds (20–25) ovate (0.2–0.3 × 0.1–0.2 mm), yellowish brown with striations.

Micromorphology of the seed exhibits a sub-orbicular shape with striations that are not parallel and cross each other towards the margin. The epidermal cell pattern is angular to spheroidal. Seed margin is entire and along the margin the cells are rectangular shaped (Fig. 7G, H).

#### Phenology.

Flowering and fruiting during August– December.

#### Etymology.

Greek psammos, sand, and philios, loving, alluding to exclusive habitat of sandy marginal zones of granite hills.

#### Habitat and distribution:

The primary habitat of *Polycarpaeapsammophila* is the hillock terrains in Palakkad district along the sandy margins. It is seen associated with *Tephrosiapurpurea* (L.) Pers., *Parasopubiadelphiniifolia* (L.) H.-P. Hofm. & Eb. Fisch. and *Glinusoppositifolius* (L.) A. DC. (Fig. 8).

#### Conservation status.

The current study is based on two different populations ranging from 50–80 individuals. We believe that further data is needed to determine the conservation status of *Polycarpaeapsammophila* because it could be found in other locations in SW-India (or India as a whole). As a result, according to IUCN criteria, the new species is classified as DD (Data Deficient) (IUCN 2021).

#### Additional specimens examined.

India. Kerala, Palakkad district, Nenmara, Vallangi, 12 September 2021, Suresh V. & Alen Alex Philip, 2065 (UCBD!); 21 September 2021, Sojan Jose & Kumar V.S.A., 2083 (UCBD!).

### ﻿ Polycarpaea barbellata

Taxon classificationPlantaeCaryophyllalesCaryophyllaceae

V.S.A. Kumar, S. Arya, V. Suresh & Alen Alex sp. nov.

4385D085-DA68-5C80-8137-1652514816CD

urn:lsid:ipni.org:names:77307991-1

Figs 5 , 6

#### Type.

India. Kerala, Palakkad district, Kuthanur, Chedukamala 10°41'42.6"N, 76°31'06.3"E, 150 m a.s.l., 20 October 2021, V.S.A Kumar, Suresh V & Arya S., 3010 (holotype UCBD! isotype UCBD!).

**Figure 5.**
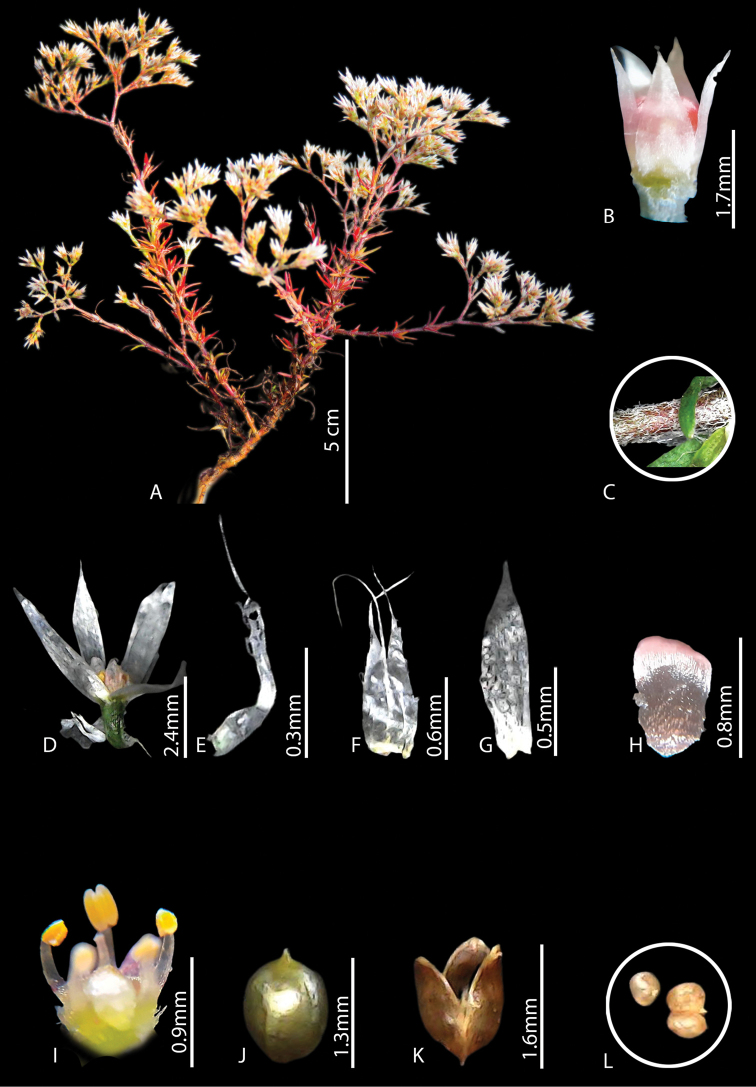
**A** habit **B** flower bud **C** internode **D** flower **E** bract **F** stipule **G** sepal **H** petal **I** stamen **J** gynoecium **K** capsule **L** seed. Photos by V.S.A. Kumar.

#### Diagnosis.

*Polycarpaeabarbellata* is morphologically similar to *Polycarpaeaaurea* with respect to yellow-orange color of sepals but differs with respect to stipules (oblong, parted into 3 with a long acicular structure in the center and other two parts barbellate *vs.* lanceolate parted into 2, free, with no central structure), Inflorescence (dense cyme *vs.* lax cyme), bract (linear, white, acicular apex *vs.* lanceolate-ovate, greyish-brown, acuminate apex), bracteoles (Capillaceous with acicular apex *vs.* ovate lanceolate with acute apex), petals (wedge shaped, whitish-lilac *vs.* broadly ovate-oblong, yellowish-brown), Gynoecium (spheroidal, yellowish green reduced *vs.* conical, short yellow), capsule (style not persistent, tip not recurved, 2–3 seeded *vs.* style persistent, tip recurved, 5–many seeded).

**Figure 6.**
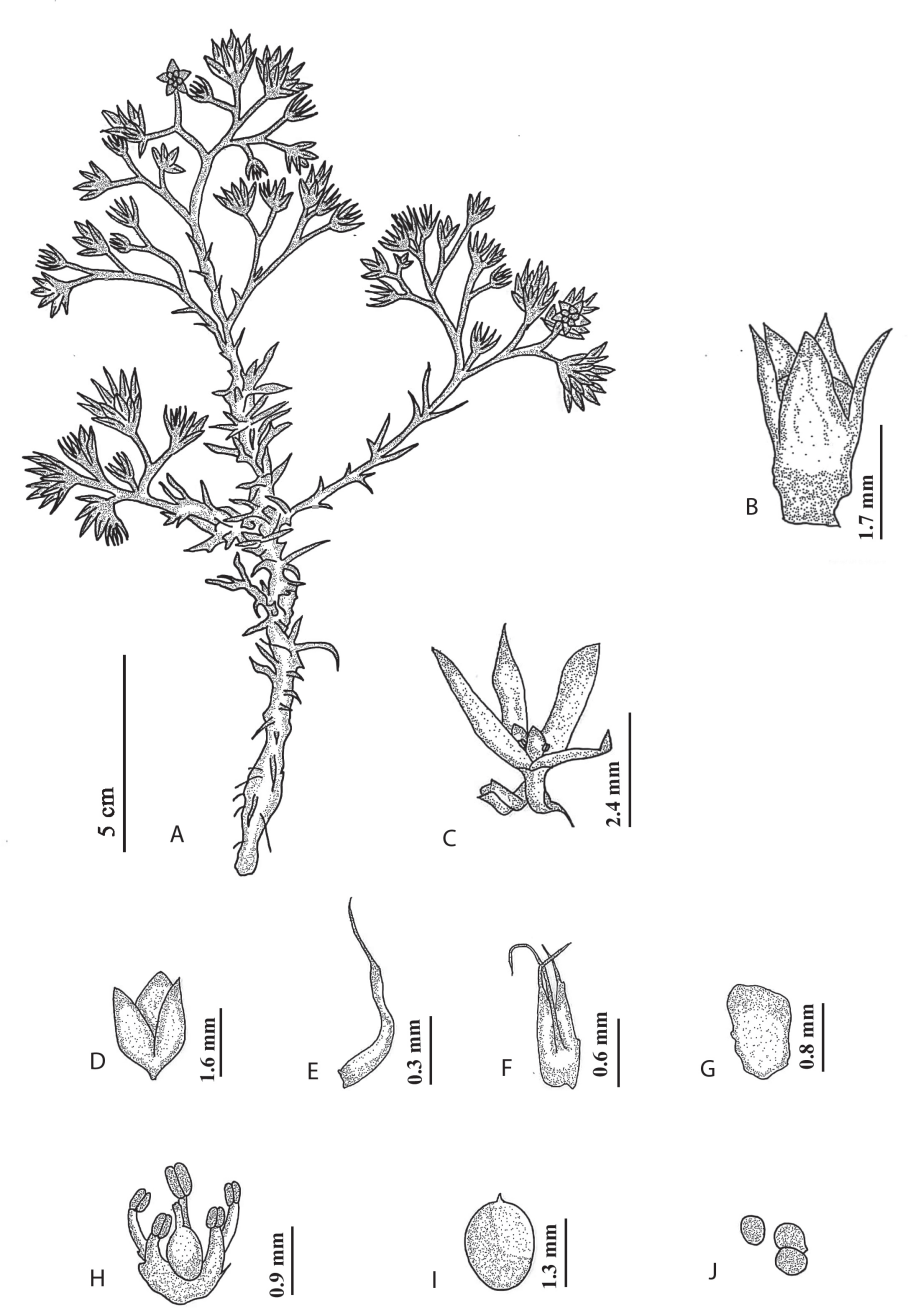
*Polycarpaeabarbellata***A** habit **B** flower bud **C** flower **D** capsule **E** bract **F** stipule **G** petal **H** stamen **I** gynoecium **J** seed. Illustration by Ambika Viswanathanpillai.

#### Description.

Annual herbs, erect or sub-erect, branched from the base 10–15 cm high. Stem terete, sparsely villous, nodes red swollen, internodes 1.5–2 cm long. Leaves whorled, sessile, lanceolate-oblanceolate, green, 1.3–2.1cm long, base round, margin smooth or wavy, apex acute or acuminate, abaxial surface glabrous, adaxial surface pubescent; lamina 1–2 veined, prominent on abaxial side; stipules prominent, oblong, parted into 3 (2 equal barbellate parts), central part has a long acicular structure ca. 1 mm long, fused at the base (1–1.2 × ca. 0.4 mm), base golden yellow, smooth; margin entire, apex acicular, milky white. Inflorescence terminal, branched regular dense cyme, ca. 4.5 cm long; Bracts linear – lanceolate, 0.8 – 1.0 mm, white, equal or sub-equal to the length of the sepal; base smooth, margin entire, apex acicular. Bracteole capillaceous with acicular apex, holding the bracts in position. Flowers 4–6 per cyme, 3.5–3.8 mm long; pedicels 1.5–2.3 mm long, green villous. Sepals 5, fused at base, ovate (1–1.2 × ca. 0.8 mm), entire at the margin, acute to obtuse at apex, white, non-membranous base round, midrib faint. Petals 5, broadly wedge shaped (0.5–1 × 0.5–0.6 mm), margin entire, completely enclosing the ovary, 1/3 as short as sepals, whitish-lilac. Stamens 5, forming a ring with petals and encircling the ovary, ca. 0.9 mm long; anthers yellow, ovate, basifixed. Ovary 1-loculed, shortly stipitate, spheroidal, yellowish green, 1.2–1.3 × 1–2 mm, glabrous, placentation free central; style 0.01–0.03 mm, shorter than the ovary; stigma capitate. Capsule oblate-prolate (1.4–1.6 × ca. 0.6 mm), shortly stipitate, style not persistent, tip not recurved, 3-valved, breaks along the suture, brownish, scarious along margin. Seeds (2–3) ovate (0.1–0.15 × 0.1–0.2 mm), yellow with striations.

**Figure 7.**
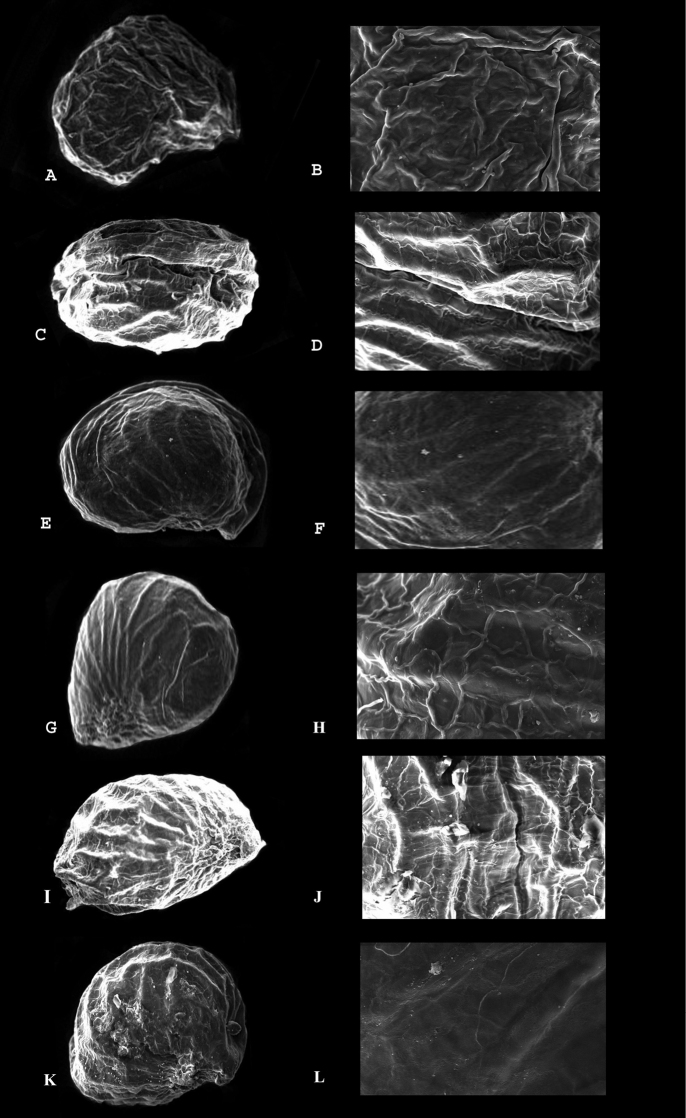
SEM of seeds (*P.corymbosa*) **A** seed **B** seed surface **C, D***P.palakkadensis***E, F***P.ebracteata***G, H***P.psammophila***I, J***P.barbellata***K, L***P.aurea*.

Micromorphology of the seed is ovate-sub-orbicular in its outline with depressions all over the seed surface. The margin is entire and the epidermal cell pattern is faintly angular. Striations are also faint (Fig. 7I, J).

#### Phenology.

Flowering and fruiting during August-December.

#### Etymology.

Latin barba, stiff hairs, suffix ella, diminutive, and -ata, possession, alluding to barbellate nature of stipules, a diagnostic character.

#### Habitat and distribution.

**Figure 8.**
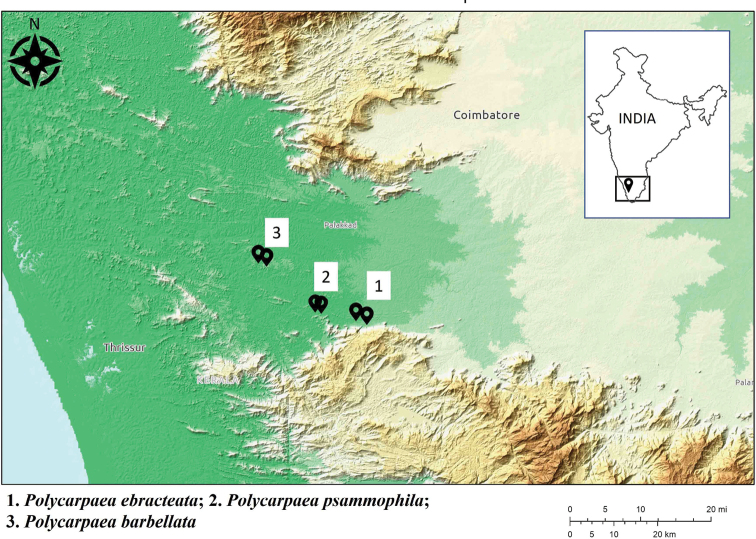
Distribution map of *Polycarpaeaebracteata*, *P.psammophila* and *P.barbellata*.

The primary habitat of *Polycarpaeabarbellata* is the hillock terrains in Palakkad district (Kerala granite outcrop in the northern side of Palakkad gap, the largest break in the Western Ghats having an arid climate with seasonal fires). *Polycarpaeabarbellata* is also seen associated with *Polycarpaeacorymbosa*, *Fimbristylis* sp. and *Indigoferaenneaphylla* (Fig. 8).

#### Conservation status.

The present study could report two populations with 20–35 individuals each. We consider that further evidence is needed to determine the new taxon’s conservation status because *Polycarpaeabarbellata* could be found in other regions in Southwest (and India as a whole). As a consequence, the new species is now categorized as DD (Data Deficient) by the IUCN (IUCN 2021).

#### Additional specimens examined.

India. Kerala, Palakkad district, Kuzhalmannam, Kariyanchirachola, 2 November 2021, Suresh V. & Sojan Jose, 3077 (UCBD!); 10 November 2021 Alen Alex Philip & Suresh V., 3084 (UCBD!).

#### Taxonomic notes.

In India, the genus *Polycarpaea* is represented by seven species (Arya et al. 2021). The proposed three new species are closely allied to *Polycarpaeacorymbosa* (Linnaeus 1753: 205) Lamarck (1792: 129) and *Polycarpaeaaurea* that has wide distribution along with *P.palakkadensis*. The new species also resembles *Polycarpaeapalakkadensis* superficially but differs distinctly with respect to characters summarized in Table 1.

**Table 1.** Morphological comparison between *Polycarpaeaebracteata*, *P.psammophila*, *P.barbellata* with *P.corymbosa*, *P.aurea* and *P.palakkadensis*.

Characters	*Polycarpaeaebracteata*	*Polycarpaeapsammophila*	*Polycarpaeabarbellata*	*Polycarpaeacorymbosa*	*Polycarpaeapalakkadensis*	*Polycarpaeaaurea*
**Leaves**	Verticillate	Whorled	Whorled	Opposite or apparently whorled	Verticillate (erroneously given as Pseudoverticillate in Protologue)	Opposite decussate
**Stipules**	Ovate-obovate, 0.2–0.8 mm, apex acute base yellowish or greenish, setae absent, white	Linear to Lanceolate, 5–8 mm, base golden yellow with unicellular setae, apex often bifurcated, branches acicular at apex, milky white	Oblong, parted into 3 (2 equal parts), central part has a long acicular structure ca 1 mm long, fused at the base 1–1.2 × ca. 0.4 mm, base golden yellow, , apex acicular, milky white	Lanceolate, long, 5 mm, base without setae, hyaline	Linear-lanceolate, 1.2 – 2 mm, base without setae, apex acute, creamy white	Lanceolate, acuminate at apex, ca. 3 mm long, base without setae, colourless or yellowish-brown, slightly silvery
**Inflorescence**	Regular branched lax cyme	Irregular branched lax cyme	Regular branched dense cyme	Irregular branched dense cyme	Irregular, dense cyme	Regular lax cymes
**Bract**	Absent	Lanceolate-oblanceolate, exceeding the length of the sepal; base smooth, margin entire, apex acuminate.	Lanceolate, equal or sub-equal to the length of the sepal; base smooth, not fused.	Lanceolate-ovate, shorter than the sepal.	Ovate-oblong (erroneously given as linear-lanceolate in protologue), fused at the base, creamy white.	Ovate-lanceolate, not fused at base, grey with a faint brownish tinge.
**Bracteole**	Absent	Linear with acicular apex	Capillaceous with acicular apex	Lanceolate with acute apex	Capillaceous, not prominent	Ovate-lanceolate
**Sepal**	Ovate 1.3–1.4mm, entire at the margin, acute or obtuse at apex, white, non-membranous base round, midrib faint.	Obovate-oblanceolate 2.3 –2.6 mm, acute or obtuse at apex, white.	Ovate 1–1.2 mm, acute to obtuse at apex, white, non-membranous base round, midrib faint.	Lanceolate, acuminate apex, hyaline, membranous.	Ovate-oblong, entire margin, acute or blunt apex, white.	Ovate-lanceolate, acute-acuminate at apex, scarious, bright orange-reddish.
**Petal**	Ovate 0.1–0.3 mm, oblong to round at apex, partially enclosing the ovary, 1/4 as short as sepals, dark red-brown.	Ovate-oblate 1–1.3 mm, keeled, pointed upward at apex, partially or completely enclosing the ovary.	Broadly wedge shaped 0.5–1 mm completely enclosing the ovary, 1/3 as short as sepals, whitish-lilac.	Broadly ovate round at apex; silvery white to pink or purplish red.	Ovate-cordate, fimbriate margin, round to mucronate at apex, dark red – brown.	Oblong-obovate, margin entire, obtuse at apex, yellowish-brown.
**Stamens**	0.2 mm long, filament inconspicuous	0.3 mm long; filament longer than anther.	0.9 mm, filament same length as anther.	2 mm, Filament equals the length of anther.	0.1 mm, filament very short	1 mm, filament as long as anther
**Gynoecium**	Spheroidal	Spheroidal	Spheroidal	Ovoid	Oblate spheroid	Conical
**Capsule**	Style not persistent ovoid 3-valved, breaks along the suture, brownish,	Style persistent, Oblate-prolate, tip not curved after dehiscence 4 valved.	Style not persistent, 3 valved, tips straight after dehiscence	Style persistent, tips not curved, 3 valved	Style not persistent, 4 valved, smooth, tips not recurved after dehiscence	Style persistent smooth, shining, glabrous, tips recurved after dehiscence
**Seed**	3–4 seeds, yellow to brown no striation	20–25 seeds yellowish brown, smooth	2–3 seed, ovate yellow with striations	5–13, brown, reniform	1–2 Ovoid-elliptical creamy white	5-many seeded, reniform brown

### ﻿A key to demarcate the new three species from the other seven species found in India

**Table:** 

1	Habitat in rocky terrains, reaching a height of 2–15cm, petal ovate – oblong, apex obtuse or round, not keeled	**2**
–	Habitat in sand, reaching a height of 18–25cm, petal ovate-oblate, apex shortly acicular, keeled	***P.psammophila***
2	Leaves radical and cauline; flowers in spike; capsule thin walled	***P.spicata***
–	Leaves cauline; flowers in dense or lax cyme; capsule thick walled	**3**
3	Bract present	**4**
–	Bract absent	***P.ebracteata***
4	Petal pinkish-purple; plant glabrous	***P.diffusa***
–	Petals pinkish-yellow or whitish-lilac or yellowish-brown; plant densely tomentose	**5**
5	Stem with greyish hairs; petal lightly coloured or hyaline; leaves set with green slender node	***P.corymbosa***
–	Stem with white hairs; petal brightly colored; leaf set with reddish swollen nodes	**6**
6	Plants not stunted; sepal bright white or red or orange; petals yellow-brown or whitish lilac	**7**
–	Plants stunted; sepal colorless; petals violet	***P.majumdariana***
7	Leaves opposite-decussate; anthers white-cream	***P.aurea***
–	Leaves pseudo whorled, whorled or verticillate, nodes red villous, anthers bright yellow	**8**
8	Stipule barbellate parted into three halves with central part acicular	***P.barbellata***
–	Stipules smooth parted into two halves with no central structure	**9**
9	Sepals ovate-oblong; petals ovate-cordate, apex round; gynoecium oblate spheroidal, capsule 1–2 seeded, seed ovoid	***P.palakkadensis***
–	Sepals lanceolate, petals ovate-lanceolate, apex acute; gynoecium prolate; capsule 3–10 seeded, seed sub-reniform	***P.rangaiahiana***

## Supplementary Material

XML Treatment for Polycarpaea ebracteata

XML Treatment for Polycarpaea psammophila

XML Treatment for Polycarpaea barbellata
